# Neurodegenerative movement disorders: An epigenetics perspective and promise for the future

**DOI:** 10.1111/nan.12757

**Published:** 2021-08-05

**Authors:** Megha Murthy, Yun Yung Cheng, Janice L. Holton, Conceição Bettencourt

**Affiliations:** ^1^ Queen Square Brain Bank for Neurological Disorders UCL Queen Square Institute of Neurology London UK; ^2^ Department of Clinical and Movement Neurosciences UCL Queen Square Institute of Neurology London UK; ^3^ Department of Neurodegenerative Disease UCL Queen Square Institute of Neurology London UK

**Keywords:** brain tissue, DNA methylation, epigenetics, epigenomics, EWAS, movement disorders, neurodegeneration, pathogenesis

## Abstract

Neurodegenerative movement disorders (NMDs) are age‐dependent disorders that are characterised by the degeneration and loss of neurons, typically accompanied by pathological accumulation of different protein aggregates in the brain, which lead to motor symptoms. NMDs include Parkinson's disease, multiple system atrophy, progressive supranuclear palsy, and Huntington's disease, among others. Epigenetic modifications are responsible for functional gene regulation during development, adult life and ageing and have progressively been implicated in complex diseases such as cancer and more recently in neurodegenerative diseases, such as NMDs. DNA methylation is by far the most widely studied epigenetic modification and consists of the reversible addition of a methyl group to the DNA without changing the DNA sequence. Although this research field is still in its infancy in relation to NMDs, an increasing number of studies point towards a role for DNA methylation in disease processes. This review addresses recent advances in epigenetic and epigenomic research in NMDs, with a focus on human brain DNA methylation studies. We discuss the current understanding of the DNA methylation changes underlying these disorders, the potential for use of these DNA modifications in peripheral tissues as biomarkers in early disease detection, classification and progression as well as a promising role in future disease management and therapy.

## Graphical abstract

This review addresses recent advances in epigenetic and epigenomic research in neurodegenerative movement disorders, with a focus on human brain DNA methylation studies. We discuss the current understanding of the DNA methylation changes underlying these disorders, the potential for use of these DNA modifications in peripheral tissues as biomarkers in early disease detection, classification and progression as well as a promising role in future disease management and therapy.

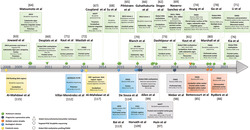

## INTRODUCTION

The ageing brain is exposed to a myriad of environmental and genetic factors that render it susceptible to certain insults and can trigger the development of several neuropathological changes, resulting in an accelerated cognitive and functional decline [1]. Neurodegenerative movement disorders (NMDs) are age‐dependent disorders characterised by the degeneration and loss of neurons, typically accompanied by pathological accumulation of different protein aggregates in the brain, leading to motor symptoms [2]. NMDs embrace a range of diseases, including Parkinson's disease (PD), atypical parkinsonisms, such as multiple system atrophy (MSA) and progressive supranuclear palsy (PSP), Huntington's disease (HD) and ataxias, such as Friedreich's ataxia (FRDA), among others [3]. Advances in research on this heterogeneous group of diseases over several decades have aided in unravelling underlying disease mechanisms and pathophysiology; however, a complete understanding of NMDs remains to be achieved.

NMDs include Mendelian disorders such as HD, FRDA and several forms of PD, which show specific inheritance patterns and result from causative mutations in single genes, as well as complex disorders, such as sporadic PD, MSA and PSP, which result from an interplay between genetic, epigenetic and environmental factors [1, 4, 5]. Noticeable heterogeneity (e.g., in the age at disease onset) is present even in Mendelian disorders, strongly suggesting the presence of other factors influencing the disease pathogenesis in addition to the causative mutations (e.g., [6, 7, 8]). NMDs, like other neurodegenerative diseases, are age dependant, and an increasing number of studies point towards epigenetic modifications as key elements in this dependency and have implicated their involvement in the aetiopathology of NMDs [9, 10, 11].

**FIGURE 1:**
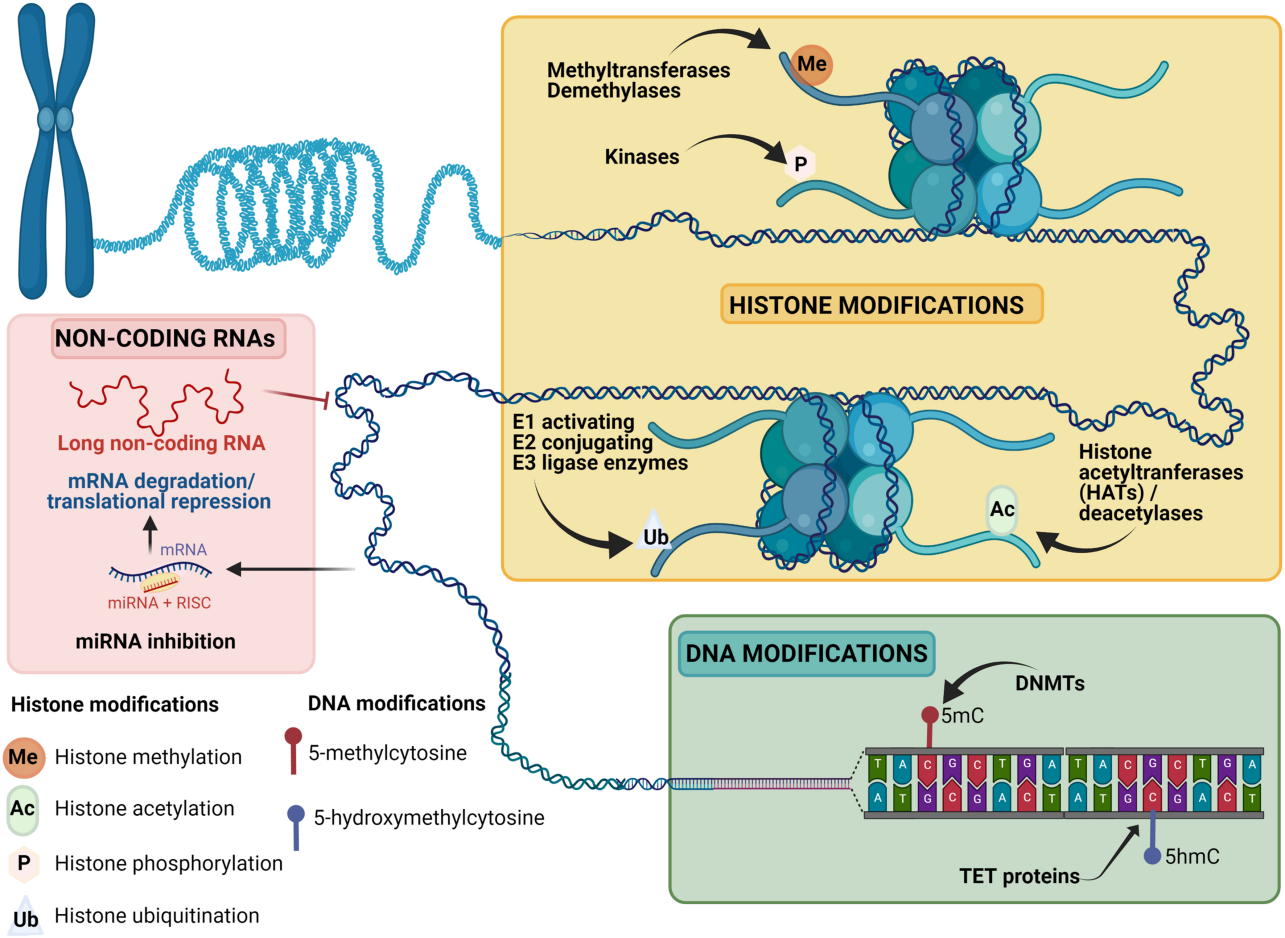
Schematic representation of the complex landscape of epigenetic mechanisms. DNA can often be modified at cytosine residues by the addition of chemical groups. Cytosines can be modified by methylation, hydroxymethylation (hmC), formylation (fC) and carboxylation (caC). Nucleosomes, which are the basic structural unit of chromatin and are composed of DNA wrapped around histone proteins, can change position to increase or decrease DNA accessibility. This DNA accessibility can be modified by the incorporation of histone variants and the addition of post‐translational modifications to histones, such as methylation, acetylation, phosphorylation and ubiquitination. Non‐coding RNAs, including microRNAs (miRNAs) and long non‐coding RNAs (lncRNAs), also play an important role in transcription regulation and in a broader definition are considered another epigenetic mechanism. While miRNAs are involved in directing RNAs for degradation, lncRNAs are associated with other complexes and can activate or repress transcription. This figure was created with BioRender.com

Epigenetic modifications are heritable but reversible changes that bring about alterations in the gene activity, or which alter gene function without changing the DNA sequence. Such epigenetic modifications/mechanisms include DNA methylation as well as histone variants and post‐translational modifications, such as acetylation, methylation, phosphorylation and ubiquitylation that can result in chromatin remodelling. Additionally, a more flexible definition of epigenetic mechanisms also includes the regulatory action of microRNAs and other non‐coding RNAs (Figure 1) [12, 13, 14, 15]. Certain epigenetic modifications, such as those involved in genomic imprinting, can be highly stable, whereas others can be dynamic and be acquired, influenced and altered by environmental factors such as diet, lifestyle, temperature changes and gene–environment interactions such as exposure to chemicals and toxins [16, 17]. Epigenetic modifications are responsible for functional regulation during development, adult life and ageing and have progressively been implicated in complex diseases such as cancer and more recently in neurodegeneration [13, 16, 18]. DNA methylation is by far the most widely studied epigenetic modification. This review will therefore summarise the recent advances in epigenetic and epigenomic research in NMDs, with a major focus on human brain DNA methylation studies. We will discuss the current understanding of the DNA methylation changes underlying these disorders, the potential for use of these DNA modifications as biomarkers in disease identification, classification and progression as well as their role in disease management and therapy.

## DNA METHYLATION AND OTHER EPIGENETIC DNA MODIFICATIONS: THEIR ROLE IN THE HUMAN BRAIN

**FIGURE 2:**
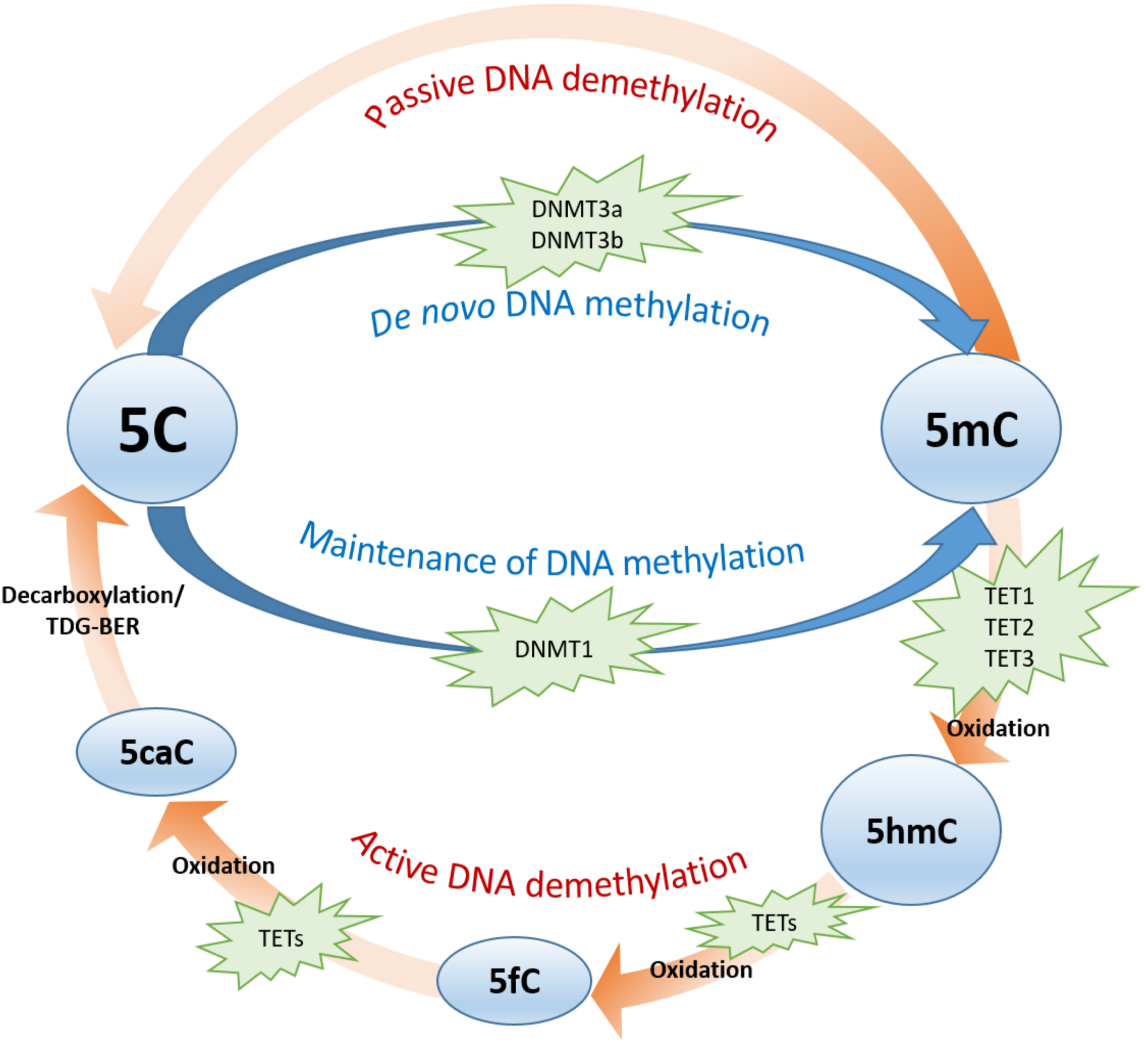
Schematic representation of the dynamic cycle for DNA cytosine modifications. Unmethylated cytosine (5C) can be converted into 5‐methylcytosine (5mC) through DNA methyltransferases (DNMTs). Active demethylation of 5mC, 5‐hydroxymethylcytosine (5hmC), 5‐formylcytosine (5fC) and 5‐carboxylcytosine (5caC) occurs via oxidation by ten‐eleven translocation (TET) enzymes and decarboxylation (?)/thymine DNA glycosylase (TDG)‐dependent base excision repair (BER)

DNA methylation is a key epigenetic mechanism regulating gene expression [19, 20]. It consists of the addition of a methyl group to the fifth carbon position of a cytosine, usually in the context of CpG dinucleotides, converting it to 5‐methylcytosine (5mC) (Figure 2). This DNA modification is brought about by a family of proteins called DNA methyltransferases (DNMTs). DNMT1 is responsible for maintaining the methylation pattern after cell division, whereas DNMT3a and DNMT3b are responsible for de novo methylation [21, 22, 23, 24, 25]. Curiously, mutations in *DNMT1* have been identified as the cause of autosomal dominant cerebellar ataxia, deafness and narcolepsy [26], which falls under the umbrella of NMDs. Genome‐wide studies report that, after the unmodified cytosine (5C), 5mC is the most common cytosine state in the brain, covering a vast majority of CpG dinucleotides, with the exception of CpG islands (high‐density CpG areas), which are frequently found at gene promoters and remain largely unmethylated [27, 28, 29, 30, 31]. Although it was initially thought that DNA methylation was exclusively associated with transcriptional repression, it has more recently been shown that the effect of this DNA modification on gene expression depends on its genomic location. While DNA methylation in gene promoters often results in a repressive effect on proximal gene expression, methylation in gene bodies is more often associated with increased expression [32, 33, 34]. In addition, DNA methylation has been demonstrated to have a functional role in modulating alternative RNA splicing, by marking exons across the genome and mediating the activities of proteins such as MeCP2 [35, 36, 37, 38, 39].

It has been shown that 5mC can be oxidised by ten‐eleven translocation (TET) proteins into a stable derivative, 5‐hydroxymethylcytosine (5hmC) (Figure 2). Although 5hmC was thought to be just an intermediate molecule prior to DNA demethylation [40], recent studies support an independent and functional role for this epigenetic mark [41]. It is interesting to note that 5hmC is enriched more than 10‐fold in the brain compared with peripheral tissues, is associated with the regulation of neuronal activity, and accumulates in the brain during neuronal development and maturation, strongly supporting a brain‐specific function for 5hmC [42, 43]. During the demethylation process, 5hmC can be further oxidised by TET enzymes into 5‐formylcytosine (5fC), which then becomes 5‐carboxylcytosine (5caC). Finally, demethylation can be completed by thymine DNA glycosylase (TDG)‐dependent base excision repair (BER) or decarboxylation, by a yet unknown decarboxylase, of 5caC forming unmodified cytosine (Figure 2) [31, 44, 45].

DNA methylation and hydroxymethylation landscapes in humans and non‐human primates show important differences in 5mC and 5hmC levels, which associate with neuronal related processes during evolution of the human brain [46]. Furthermore, studies in healthy human brain tissue [47, 48] strengthen the hypothesis that DNA methylation/hydroxymethylation patterns display temporal and regional specificity. Such specificity transcends brain regions, as there are differential roles for 5mC and 5hmC in regulating gene expression in different brain cell types, adding additional layers of complexity that need to be addressed when designing experiments to identify disease‐specific DNA methylation dysregulation [49].

## WHAT DO WE KNOW ABOUT BRAIN GLOBAL OR LOCI‐SPECIFIC DNA MODIFICATIONS IN NMDS?

**FIGURE 3:**
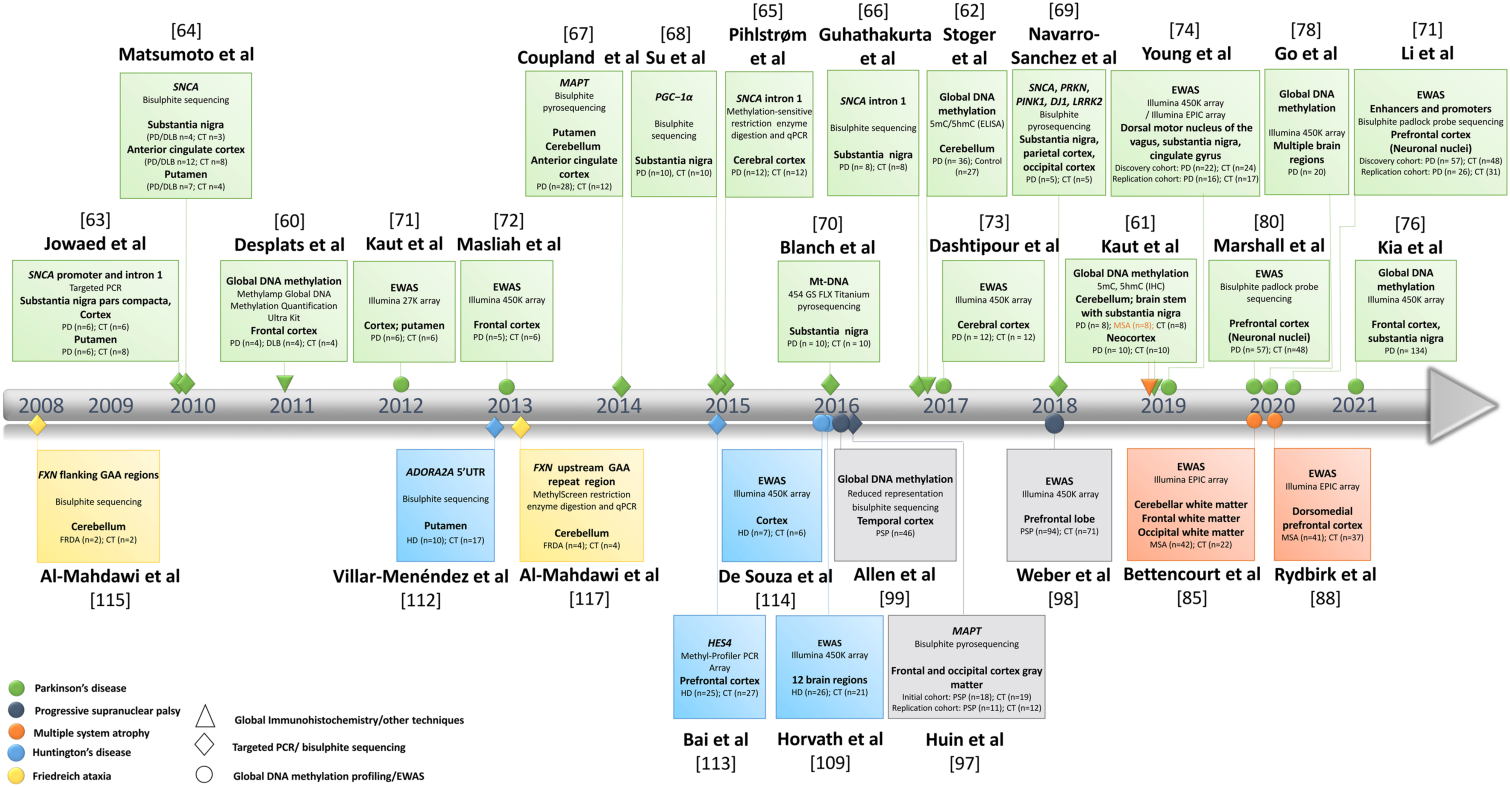
A timeline summarising the progress of studies investigating DNA modifications in brain tissue of neurodegenerative movement disorders (NMDs). Different colours represent different NMDs and different shapes represent different methodological approaches and study designs, as depicted in the figure caption. DLB, dementia with Lewy bodies; EWAS, epigenome‐wide association study; FRDA, Friedreich's ataxia; HD, Huntington's disease; MSA, multiple system atrophy; PD, Parkinson's disease; PSP, progressive supranuclear palsy; 5mC, 5‐methylcytosine; 5hmC, 5‐hydroxymethylcytosine

An increasing number of studies have reported DNA methylation changes in neurodegenerative diseases, some of which investigated global methylation patterns, whereas others focused on candidate genes. More recently, accompanied by technological advances in the field, most studies queried genome‐wide DNA methylation changes at single nucleotide resolution. Most studies, however, have analysed total methylation patterns (5mC + 5hmC), and only a few have investigated 5mC and 5hmC individually. Within neurodegenerative diseases, the role of DNA methylation has been most extensively studied in Alzheimer's disease (AD) and has been reviewed elsewhere (e.g., [50, 51]); therefore in the following section, we will focus on studies utilising brain tissue from NMDs, including PD, MSA, PSP, HD and FRDA (Figure 3). For a more comprehensive overview of the biological significance of the results discussed, we provide a summary table of differentially methylated genes that have been identified in two or more studies, both within an NMD or between different NMDs (Table S1).

### Parkinson's disease and other Lewy body diseases

PD, the second most common neurodegenerative disease, is an adult‐onset progressive movement disorder, which presents with motor symptoms such as tremor, rigidity, bradykinesia and postural instability, as well as numerous non‐motor symptoms. The symptoms of PD manifest due to a lack of dopamine in the basal ganglia, which is caused by the death of dopaminergic neurons in the substantia nigra pars compacta [52]. Pathological hallmarks of PD include cytoplasmic aggregates of α‐synuclein within neurons, mostly in the form of Lewy bodies [53]. PD is often accompanied by cognitive impairment, classified as PD dementia, whose symptoms overlap with dementia with Lewy bodies (DLB), the second most common form of dementia after AD [54]. DLB is a complex disorder characterised by parkinsonism, cognitive fluctuation and visual hallucinations [55, 56]. Rare variants in more than 20 genes, including *SNCA* and *LRRK2*, have been reported to cause PD, and more than 90 PD risk loci have been identified in recent large genome‐wide association studies, as reviewed elsewhere [57]. The strong phenotypic overlap between PD and DLB is also reflected in the genetics of DLB [58], with established risk factors, such as *SNCA* and *GBA* also being implicated in PD. Notwithstanding, the aetiology of PD and DLB is complex, entailing numerous genetic, epigenetic and environmental factors [59]. Regarding epigenetic factors, we detail below studies reporting changes in DNA modifications mostly in PD, as studies focusing on DLB are practically non‐existent.

Global DNA methylation analysis using immunodetection of 5mC showed at least a 2‐fold reduction in 5mC immunoreactive nuclei, indicating global loss of methylation in PD and DLB frontal cortices compared with controls [60]. More recently, Kaut and colleagues, using qualitative and semi‐quantitative analysis of 5mC and 5hmC, found 5mC to be significantly increased in the cortex of PD patients, contrasting with the results from the previous study, whereas 5hmC was only elevated in the cerebellar white matter [61]. Another examination of 5mC and 5hmC in the cerebellum of PD samples, using enzyme linked immunosorbent assay (ELISA), showed extensive inter‐individual variation in 5mC in both PD and controls, but the range of 5hmC levels was significantly higher in PD than in controls, similar to the previous study [61, 62]. Overall, these studies have failed to reach consensus regarding the occurrence of global DNA methylation changes in PD and DLB.

Gene‐based studies have also been carried out in PD. Hypomethylation in intron 1 of *SNCA* in multiple brain regions, including the substantia nigra, has been described in several relatively small studies [60, 63, 64, 65]. These reports seem to suggest that demethylation in *SNCA* leads to overexpression of α‐synuclein, triggering its neuronal aggregation, and ultimately resulting in PD. However, another study of similar size failed to show hypomethylation in *SNCA* [66], raising caution in interpreting such findings. Other loci have also been explored, including the *MAPT* promoter region [67], which showed hypermethylation in the cerebellum and hypomethylation in the putamen in PD when compared to that in controls. Hypermethylation of the *PGCα‐1* promoter has also been reported in the substantia nigra of PD cases compared to controls, with a corresponding decrease in the mRNA and protein expression levels [68]. In addition, hypomethylation of *PRKAR2A* promoter has also been reported [60]. A pilot study, in the substantia nigra of PD and controls, investigating methylation patterns in promoter regions of *SNCA*, *PRKN*, *PINK1, DJ‐1* and *LRRK2* suggested distinct methylation patterns at a few positions in PD samples [69]. The mitochondrial DNA methylation pattern has also been studied in the substantia nigra of PD and control individuals and a loss of methylation in nearly all CpG and non‐CpG sites in the D‐loop region was reported in PD [70].

The first epigenome‐wide association study (EWAS) investigating DNA methylation landscapes in the putamen and cortex of PD and controls revealed three differentially methylated genes, including hypomethylation (>2.5‐fold) of cytochrome P450 2E1 gene (*CYP2E1*), which is predominantly expressed in neurons; a corresponding increase in its mRNA expression levels in both the putamen and cortex of PD patients was also reported, suggesting that altered methylation in cytochrome might cause an increase in susceptibility to PD [71]. Other EWAS have followed, and Masliah et al. [72] identified 2908 differentially methylated regions in PD frontal cortex. Another EWAS in the cerebral cortex of PD and controls also reported a high number of differentially methylated regions (DMRs) in PD. Notwithstanding, this study highlights significant hypermethylation of synphilin‐1, α‐synuclein‐interacting protein (*SNCAIP*) gene, suggests that *SNCAIP* might be downregulated in individuals with PD and proposes that *SNCAIP* hypomethylation could have a protective effect against developing PD [73]. A more recent and larger genome‐wide DNA methylation study in the dorsal motor nucleus of the vagus (DMV), substantia nigra and cingulate gyrus in PD cases and controls identified, in a joint analysis of discovery and replication cohorts, 234 significant DMRs in the DMV, 44 in the substantia nigra and 141 in the cingulate gyrus. A DMR in the promoter of *LOC100420587*, an SHC binding and spindle associated one pseudogene, was significant in all three regions. Other DMRs that were commonly identified in at least two regions included six genes (*NXN*, *ZFPM1*, *MAP1A*, *PRDM16*, *SPON1* and *HLX*) in the DMV and cingulate gyrus, six genes (*SBNO2*, *SLC12A7*, *DUSP22*, *ARFGAP1*, *KCNQ1DN* and *IFT140*) in the DMV and substantia nigra and eight genes (*REC8*, *HIF3A*, *APBB2*, *NDRG4*, *RARA*, *LINC00636*, *SLFN12* and *IQSEC1*) in the substantia nigra and cingulate gyrus. Functional enrichment analysis of those DMRs points towards perturbation in the Wnt and Hippo signalling pathways in PD [74]. Given the impact of these pathways in neuronal cell fate and functioning as well as in neuroinflammation, Wnt and Hippo signalling associated proteins may play an important role in PD [74, 75]. A recent multiomics study uncovering the mechanisms through which genetic risk variants are associated with PD risk, reinforced a mechanistic role for DNA methylation in modulating disease risk by regulating expression or splicing for *GPNMB*, *TMEM163* and *CTSB*, for example [76].

In an attempt to identify changes shared across neurodegenerative diseases, Sanchez‐Mut and colleagues performed DNA methylation analyses of PD, DLB, AD and Down's syndrome, using whole‐genome bisulphite sequencing (WGBS) and other technologies. WGBS identified 709 differentially methylated regions shared across all diseases, which were enriched for genes involved in synapse related pathways as well as genes associated with the regulation of cytoskeleton, neurotrophin and ErbB signalling and developmental networks such as those associated with the Wnt and Hippo pathways [77].

As a way of dissecting the involvement of DNA methylation changes in PD risk due to exposure to environmental factors, Go and colleagues performed an EWAS in the temporal cortex of Japanese immigrant plantation workers with PD, who were exposed to pesticides (organochlorines) and controls. Among the top most‐significant hypermethylated loci were CpGs mapping to prostaglandin D2 synthase (*PTGDS*) and peroxisomal biogenesis factor 19 (*PEX19*), when comparing PD individuals with 10+ years plantation work exposure to those with no exposure. Enrichment analysis of DMRs showed ‘neurological disease; cell development, survival and death; and nervous system development and function’ among the top pathways. In a combined analysis of brain and blood, this study highlighted significant association with genes such as *AIG1*, *GRIN2A*, *BCL2*, *SGK1* and *MAPT*. The study showed that DMRs in the samples differed with the exposure time to pesticides, demonstrating the dynamic effect of environmental toxins in the methylation profiles that can exacerbate or increase the risk of PD [78].

DNA methylation patterns can vary in a cell type‐specific manner, which may not be detectable when analysing bulk brain tissue. Studies enriching for specific cell types have been helpful in dissecting those changes [79]. Using such a strategy, a recent study investigated genome‐wide DNA methylation and hydroxymethylation patterns of over 30,000 brain enhancers in the neuronal nuclei isolated from the prefrontal cortex of patients with PD and controls, using targeted bisulphite deep‐sequencing [80]. This study identified 1799 significant differentially methylated cytosines at the enhancers in PD neurons compared with controls, most of which were hypermethylated; this finding was verified in an independent cohort of patients with PD and controls. Apart from investigating methylation patterns at CpG sites, this study has also studied CpH (H = A, T or C), and found that CpA sites were the most frequently disrupted at enhancers in PD. Differentially methylated enhancers affected neurogenesis, neurodevelopment and synaptic structure pathways. This study highlights the epigenetic disruption of an enhancer targeting *TET2*, a key regulator of DNA methylomes (Figure 2), as well as dysregulation in enhancers of PD risk genes such as *DNAJC6*, *DCTN1*, *PRKN*, *PLA2G6* and *FBXO7*. By comparing the early and late PD stages (Braak stage 3–4 and Braak stage 5–6, respectively), findings suggest a prominent dysregulation of enhancers in PD neurons even before the onset of Lewy body pathology, including DNA methylation abnormalities at *TET2* and *PRKN* enhancers [80]. This study shows that the widespread increase in cytosine modifications at enhancers in PD neurons coincides with a gain in hydroxymethylation accompanied by upregulation of *TET2*. Interestingly, functional in vitro and in vivo investigations support a neuroprotective effect of reduction of *TET2* in neurons [80]. Another interesting finding by the same research group is the hemispheric DNA methylation asymmetry associated with symptom lateralization in PD, mainly affecting genes involved in neurodevelopment, immune activation and synaptic transmission [81].

### Multiple system atrophy

MSA is a rare, predominantly sporadic, nervous system disorder of adult‐onset. The presence of glial cytoplasmic inclusions (GCIs) in oligodendrocytes, along with progressive neurodegeneration, is the neuropathological hallmark in MSA [82]. The identification of α‐synuclein as a major component of the GCIs established the first link between MSA and PD and DLB [83]. MSA shows differences in both clinical and pathological presentation, with the clinical presentation consisting of either MSA with predominant parkinsonism (MSA‐P) or MSA with predominant cerebellar ataxia (MSA‐C), and pathological presentations such as striatonigral degeneration (SND) or olivopontocerebellar atrophy (OPCA), or both (SND = OPCA) [84]. The existence of geographical variation in the predominance of the MSA pathological subtypes may reflect, at least in part, an interplay between genetic and environmental factors possibly through modulation of epigenetic mechanisms in MSA [84].

Qualitative and semi‐quantitative analysis of global DNA methylation levels using 5mC and 5hmC as markers demonstrated identical patterns of immunoreactivity in most parts of the brain of healthy controls and patients with MSA. However, changes in specific parts of the cerebellum were found, such as lower 5mC levels in the MSA granule cell layer, and higher percentage of 5hmC‐immunoreactive cells in the MSA cerebellar white matter when compared with controls [61]. Despite the technical limitations of this type of study, including undocumented pathological subtypes of the MSA patients studied, these findings support cell type‐specific vulnerability to epigenetic changes in MSA [84].

More recently, genome‐wide DNA methylation studies have been conducted to understand DNA methylation changes involved in MSA and their role in disease pathogenesis in different brain regions. The first MSA EWAS was conducted by our group to investigate alterations in methylation patterns in post‐mortem white matter tissue from cerebellum, frontal lobe and occipital lobe. We used a multiphase study design and included the three main pathological MSA subtypes (MSA mixed, MSA OPCA and MSA SND) as well as healthy controls [85]. Among the most consistently differentially methylated loci, across brain regions and pathological subtypes, were CpGs mapping to *MOBP*, *HIP1* and *LMAN2*. The expression of *MOBP* mRNA and other myelin related genes is indeed downregulated in MSA cerebellar white matter as well as in laser captured oligodendrocytes [86], and we have shown this downregulation is likely to be driven by hypermethylation at the *MOBP* promoter region [87]. More interestingly, a follow‐up study of a few EWAS hits led to the discovery that MOBP and HIP1 proteins are mislocalised into the GCIs, where they interact with α‐synuclein, emphasising the relevance of these loci in MSA [87]. In our genome‐wide DNA methylation analysis [85], we also identified 45 highly correlated CpG clusters using weighted gene co‐expression network analysis (WGCNA). One cluster containing a CpG in *SNCA* (cg15402943) displayed a strikingly strong negative correlation (*r* = −0.91) with the MSA disease status (i.e., pathological MSA diagnosis as compared with controls), indicating that this cluster of CpGs shows significantly lower levels of methylation in MSA compared with that in controls. MSA subtype‐specific and MSA neurodegeneration‐associated DNA methylation cerebellar signatures were also identified and implicated neurodegeneration relevant pathways, including cell death signalling, Rho GTPase signalling, axon–oligodendrocyte coupling, protein quality control, neuroinflammation and mitophagy [85].

Another recent EWAS conducted by Rydbirk and colleagues [88] investigated both 5mC as well as 5hmC in the prefrontal cortex (mildly affected area) of MSA samples and controls. The study identified five differentially methylated positions in the 5mC fraction, of which two mapped to *AREL1* and *KTN1* genes, while the others were intergenic. In the *AREL1* region, a reduction of 5mC (Δβ = −9.1%) and an increase in 5hmC was observed (Δβ = 8.5%). *AREL1* is related to antigen presentation, and the observed changes in *AREL1* were accompanied by increased gene expression of MHC Class I HLAs, further implicating antigen presentation as a disease factor in MSA. This study also reports several differentially methylated regions, including a region in the *FUT4*/*PIWIL4* promoter, which had also been observed in our EWAS data [89], replicating our previous findings. However, our study did not detect any changes in *AREL1*. The marked differences in the study designs of the two MSA EWAS are likely to be the basis of such discrepancies. Bigger unified studies across brain regions and covering all MSA subtypes may help to elucidate and catalogue the epigenome‐wide methylation profile in MSA.

### Progressive supranuclear palsy

PSP is a rare, sporadic, adult‐onset, progressive neurodegenerative disease that involves a range of motor abnormalities such as postural instability, supranuclear vertical gaze palsy, akinesia and bradykinesia, as well as cognitive and behavioural impairments [90, 91]. PSP is a form of atypical parkinsonism pathologically characterised by the intracellular aggregation of 4‐repeat (4R) tau protein in the form of neurofibrillary tangles, neuropil threads, oligodendroglial coiled bodies and tufted astrocytes in the subcortical and cortical regions of the brain, resulting in neurodegeneration [92, 93]. PSP is a complex disease, with genetic, environmental and epigenetic factors thought to contribute to the disease onset and progression. Several loci have been associated with the disease risk, including *MAPT* as the most significant risk locus, and several other genes, such as *STX6*, *EIF2AK3*, *MOBP, SLCO1A2* and *DUSP10* [94, 95, 96].

Similar to other NMDs, research into the identification of nature and role of epigenetic contributors in the brains of PSP patients has been fairly recent and scant. DNA methylation assessment in the brains was initially conducted in samples of the frontal and occipital cortices of PSP and control individuals, by pyrosequencing five CpGs in *MAPT* in a candidate gene approach. Hypomethylation of one CpG island site was observed in the frontal cortex of the PSP group, a brain region characterised by neurofibrillary degeneration. This result remained significant upon replication in an independent cohort of PSP and controls [97].

More recently, an EWAS conducted by Weber and colleagues in the prefrontal lobe tissue of PSP and control individuals, detected 627 significantly hypermethylated and 90 hypomethylated regions, the majority of which were associated with protein coding genes (62% hypermethylated and 70% hypomethylated). Strikingly, no significant DNA methylation changes were observed in *MAPT* [98]. As a major finding, this study highlights a significant increase in methylation at multiple CpG sites in *DLX1*, which is a transcription factor mainly expressed in the GABAergic inhibitory interneurons and known to play major roles in their development and function. In vitro studies of *DLX1* and the overlapping anti‐sense *DLX1AS* gene revealed that overexpression of *DLX1* results in downregulation of *MAPT* whereas overexpression of *DLX1AS* leads to upregulation of *MAPT*, leading to the hypothesis that this locus contributes to the pathogenesis of PSP by influencing *MAPT*. From a different perspective, Allen and colleagues investigated DNA methylation in PSP brain tissue to dissect whether the mechanism behind genetic PSP risk variants is related to DNA methylation changes, indeed revealing several associations in the vicinity of PSP associated loci, including *MOBP* [99].

### Huntington's disease

HD is the most common polyglutamine (polyQ) disorder, characterised by motor impairment and the presence of involuntary jerky movements of the face and limbs, termed chorea, accompanied by personality changes, weight loss and cognitive disorders [100]. HD is an autosomal dominant, fatal disease, caused by an aberrant CAG trinucleotide repeat expansion (>36) in the huntingtin (*HTT*) gene [101, 102]. The repeat expansion results in the formation of a misfolded HTT protein, which is cleaved, and forms intracellular aggregates in the cell nucleus, cytoplasm, neurites and terminals resulting in the degeneration of neurons, preferentially in the caudate nucleus and the putamen [100, 103]. The age of onset of motor symptoms shows significant inverse correlation with the number of CAG repeats [104]. However, the length of the trinucleotide repeat accounts for only 50–70% of the variability in the age of onset [105]. The heterogeneous disease presentation in terms of onset and severity, even when the number of CAG repeat expansion is identical, supports the influence of other genetic, epigenetic or environmental factors in HD [6, 106].

Researchers have investigated the epigenetic changes taking place in HD. Historical publications, which predate the identification of the *HTT* CAG repeat expansion in HD, had already suggested DNA methylation changes associated with the disease locus [107, 108]. It is interesting to note that, many years later, hypermethylation at cg22982173 in exon 1 of *HTT* was reported as the most significantly associated CpG with the HD status [109, 110]. Candidate gene approaches have been applied based on genes that show transcriptional dysregulation in HD [111], revealing increased 5mC levels and reduced 5hmC in the 5′UTR region of *ADORA2A* in the putamen of HD patients with respect to controls [112]. In addition, a gene‐specific DNA methylation analysis was conducted for *HES4* in the prefrontal cortex of HD cases and controls following the identification of loss of trimethyl‐histone H3‐lysine 4 (H3K4me3) at the *HES4* promoter in HD. This study revealed hypermethylation at the *HES4* promoter and a resultant alteration in the expression of *HES4* as well as *MASH1* and *P21*, which are downstream target genes of *HES4*, and have been shown to be involved in striatal development [113].

Almost simultaneously, two brain HD EWAS were published in 2016 [109, 114]. The study by De Souza et al. [114], likely due to the small sample size, failed to identify differential methylation associated with the HD status. The study by Horvath et al. [109], however, with data generated for 475 brain samples from various brain regions (i.e., the frontal, occipital and parietal lobes) of HD cases and controls reports 1467 CpGs, including CpGs in *TMEM8A*, *IDE* and *GRIK2*, to be significantly associated with HD in a meta‐analysis comprising the three brain regions analysed.

### Friedreich's ataxia

FRDA is an autosomal recessive ataxia, mainly caused by a homozygous (GAA)n repeat expansion in the *FXN* gene [5]. The expansion results in the reduced expression of frataxin in the cerebellum and dorsal root ganglia leading to neurodegeneration and symptoms of ataxia, dysarthria, muscle weakness and sensory loss, in addition to cardiomyopathy and diabetes [115, 116]. Studies investigating brain DNA methylation in FRDA and other ataxias are scarce and for FRDA, to the best of our knowledge, only a few small candidate gene studies covering the *FXN* gene were conducted in brain tissue. DNA methylation analysis in three regions of *FXN* in FRDA patients and controls using targeted bisulphite sequencing reported hypermethylation in a region upstream of the GAA repeat expansion in *FXN* in both brain and heart tissues, which could be responsible for the inhibition of *FXN* expression FRDA patients, while CpG sites downstream of the *FXN* GAA repeat were hypomethylated [115]. At the *FXN* promoter, although changes were detected in heart tissue, DNA methylation differences were minimal in brain tissues of FRDA patients as compared with that in controls, suggesting tissue specificity in terms of methylation differences [115]. Hypermethylation of CpG sites upstream GAA repeat in FRDA tissues as compared with controls was replicated in a follow‐up study [117]. It was also reported that FRDA cerebellum tissue displays an increase in 5hmC‐modified DNA rather than 5mC in the region upstream the *FXN* GAA repeat [117]. Epigenome‐wide approaches remain to be applied to FRDA as well as to other rare NMDs, including many cerebellar ataxias and corticobasal degeneration, for example.

## ARE THERE SIGNS OF EPIGENETIC AGE ACCELERATION IN NMDS?

Ageing is an important risk factor for neurodegeneration, and studies have revealed that the process of ageing involves several key epigenetic modifications, which are influenced by several factors that include physiological changes as well as environmental stresses or stimuli [16]. Over the last decade, several methods have been developed to estimate the biological age of an individual based on the DNA methylation states of specific sites, referred to as ‘epigenetic ageing clocks’ [118, 119, 120, 121]. These clocks have been widely used to identify differences between chronological age and biological age, that is, epigenetic age acceleration, in health and disease, including neurodegeneration. Each epigenetic ageing clock has its strengths and limitations considering their utility in investigating neurodegenerative diseases, which have been reviewed elsewhere [122].

Although studies investigating epigenetic age acceleration in brain tissue of NMDs are scarce, there is some support for age acceleration in NMDs. In an HD study, epigenetic age acceleration in specific brain regions (frontal lobe, parietal lobe and cingulate gyrus) was significantly associated with the disease status. In addition, epigenetic age acceleration was inversely correlated with the CAG repeat length of *HTT* in HD brain samples and positively correlated with the age of onset of motor symptoms [109]. Large studies using HD blood samples have also reinforced the idea of increased epigenetic age in HD (e.g., [110]). Although no studies investigated age acceleration in brain tissue of PD, this phenomenon has been observed in PD blood [123, 124]. Studies covering other NMDs are lacking.

## EPIGENETIC DNA MODIFICATIONS BEYOND THE BRAIN: OVERCOMING LIMITATIONS AND RAISING POTENTIAL

Analysis of DNA methylation in disease brain tissue has certainly helped researchers attain a more comprehensive understanding of molecular alterations taking place in the brain and the mechanisms behind such changes in NMDs. However, owing to the lack of brain tissue accessibility in life, such studies have several limitations. Molecular alterations in the brain cannot currently be used as disease biomarkers for diagnosis, prognosis and/or for evaluating drug efficacy. In addition, longitudinal studies investigating an individual over multiple time‐points, which could appropriately assess disease progression, are not feasible. Hence, several groups have attempted to identify DNA methylation signatures in more easily accessible tissues, such as blood, cerebrospinal fluid (CSF) or saliva, which could be valuable in monitoring the disease‐related changes in NMDs [55, 125, 126, 127, 128, 129, 130, 131]. Ideally, one would like to find concordant signatures (i.e., proxies) between the brain and easily accessible peripheral tissues, and a few studies have attempted this type of approach. As an example, Masliah and colleagues [72] identified 2908 DMRs in the PD frontal cortex and 3897 DMRs in the blood of PD samples; both blood and brain showed overall similar methylation patterns, with a majority of the probes showing hypomethylation and ~30% of the annotated genes showing concordant changes in methylation in both blood and brain. *HLA*‐*DQA1*, *GFPT2*, *MAPT* and *MIR886* were reported to show significant common differential methylation in both blood and brain and have also been previously implicated in PD pathogenesis, suggesting the potential of DMRs in peripheral blood leukocytes to be used as biomarkers in PD. As another example, the largest DNA methylation study conducted in HD by far, involving the characterisation of DNA methylation levels in over 2000 human samples [110], shows hypermethylation at CpG cg22982173 in exon 1 of *HTT* as the most significantly associated with HD status. This finding was validated in the brain using 475 brain methylation data from a previous study by the same group [109], suggesting for the first time, that a correlation might exist in the methylation pattern in HD between the blood and the brain. This type of approach may not always be achievable for multiple reasons, such as limited availability of brain tissue samples and/or lack of concordance between the data obtained from brain tissue and that from peripheral tissues due to tissue specificity. However, peripheral tissue DNA methylation signatures could per se be valuable, as discussed next.

As for other neurodegenerative diseases, the pathogenesis of NMDs begins years before clinical symptoms. Hence, early disease detection and interventions will be key to minimise neuronal loss, maintain brain function and prevent/reduce symptoms. Identifying early DNA methylation changes, occurring at the pre‐symptomatic or prodromal stage, would therefore be valuable. In HD, where the pre‐symptomatic stage can be clearly defined by the absence of symptoms in carriers of the disease mutation, a few studies have been conducted. An EWAS in a cohort consisting of pre‐symptomatic and symptomatic HD mutation carriers, and healthy controls, has shown promising results by identifying significant DNA methylation changes in *CLDN16*, *DDC* and *NXT2* in the pre‐symptomatic HD individuals compared with controls, but not upon the comparison of symptomatic patients with controls [132]. Another study identified an increase in *BDNF* promoter methylation observed in the blood of HD mutation carriers (pre‐symptomatic and symptomatic), which correlated with psychiatric symptoms in HD, suggesting that it may be relevant as a biomarker in HD [125]. For more complex NMDs, such as PD, MSA and others, it is certainly more challenging to define this pre‐symptomatic or prodromal stage, and appropriate DNA methylation studies are lacking. To improve our understanding of disease progression in NMDs as well as to monitor the effects of treatments over time, there is a need for appropriately designed longitudinal studies. With this in mind, a relatively recent longitudinal PD EWAS reveals genomic sites showing longitudinal DNA methylation changes associated with disease progression as well as methylation changes in response to dopaminergic medications in the blood methylome [126]. Hopefully, additional longitudinal studies will be performed to appraise the utility of DNA methylation signatures as tools for early disease detection, monitoring disease progression and determining the beneficial effects of drugs in NMDs.

As many NMDs overlap in their clinical presentation, a definitive diagnosis can only be made by the post‐mortem examination of the brain. This is the case for PD and the atypical parkinsonisms, such as MSA and PSP, for example, and often leads to misdiagnosis [133, 134]. The integration of DNA methylation profiles into the classifiers of central nervous system tumours, which includes over 100 subtypes, has significantly improved the accuracy of the diagnosis [135, 136]. Therefore, the possibility of diagnosing NMDs in life with greater accuracy by making use of DNA methylation footprints holds great potential. Indeed, efforts to integrate DNA methylation in NMD classifiers are starting to emerge, such as the study by Wang and colleagues [131], which identified a blood‐based 53‐gene signature that seems to distinguish PD patients from healthy controls. However, well‐designed studies encompassing large cohorts across NMDs to identify disease‐specific signatures are needed [137].

## DNA METHYLATION‐BASED THERAPIES: OPENING UP POSSIBILITIES

As DNA methylation is reversible, it may provide avenues for treatment. Therapies based on DNA methylation have been proposed for several diseases and comprise the use of demethylases, DNMT inhibitors (DNMTi's) as well as gene‐editing based therapies. Some DNA demethylating drugs and DNMTi's have already been approved for the treatment of certain types of cancer [138], and the applicability of these strategies to neurodegenerative diseases is being investigated [137, 139]. As it has been reported that α‐synuclein sequesters DNMT1 from the nucleus into the cytoplasm in neurons, likely inducing loss of DNMT1 nuclear function [60], and it has also been shown that the reduction of TET2 in neurons could be neuroprotective in PD [80], these two DNA methylation machinery enzymes may be good therapeutic targets for neurodegeneration. Although promising, approaches targeting the DNA methylation machinery would have global effects on methylation levels and may therefore have unwanted side effects. Hence, it is imperative that we design epigenetic therapies involving (a) small‐molecule epigenetic modulators acting as pharmacological modifiers of site‐specific DNA methylation status and (b) appropriate drug delivery systems that act as vehicles carrying these modulators preferably across the blood–brain barrier. In this respect, exciting methods for in vivo site‐specific manipulation of DNA methylation have been designed using deactivated endonuclease Cas9 fused to enzymes that methylate or demethylate DNA specific sites [140]. CRISPR‐based tools also allow for efficient in vivo targeting of many different sites using multiple guide RNAs (sgRNAs) to alter the methylation status at different genomic locations simultaneously [141]. One such all‐in‐one lentiviral vector targeting DNA methylation in *SNCA* intron 1 was designed by fusing CRISPR‐deactivated Cas9 (dCas9) with the catalytic domain of DNA‐methyltransferase 3A (DNMT3A) has been applied to human induced pluripotent stem cell (hiPSC)‐derived dopaminergic neurons from a PD patient with the *SNCA* triplication, which successfully resulted in downregulation of *SNCA* mRNA and protein [142]. A translation of such potential targets in vivo can be a possible strategy for precision therapy in PD and other NMDs.

## CONCLUDING REMARKS

Extraordinary progress has been made in recent years to identify DNA methylation changes occurring in the brains of NMDs. A systematic unbiased comparison of brain DNA methylation changes between studies of NMDs is challenging owing to the wide range of identification techniques and the brain regions analysed. In addition, most of the studies have relatively small sample sizes, resulting in lower statistical power to detect significant differential methylation. This is compounded by the effect sizes (differences in methylation levels) in NMDs and other neurodegenerative diseases being smaller than those identified in other diseases such as cancer.

There is much to be explored, and well‐powered brain and peripheral tissue studies covering a more comprehensive number of NMDs are still needed, especially for rarer NMDs, such as corticobasal degeneration and many cerebellar ataxias. Achieving a broader understanding of the role of DNA methylation across NMDs has great potential as it might allow the identification of (a) disease‐specific signatures that could improve diagnostic accuracy, early disease detection and follow‐up of progression, and (b) cross‐disease DNA methylation changes that could set common targets for drug development across several NMDs and perhaps other neurodegenerative diseases. Excitingly, recent site‐specific DNA methylation editing approaches raise new avenues for targeted therapeutic design.

## CONFLICT OF INTEREST

The authors declare that they have no conflict of interest.

## AUTHOR CONTRIBUTIONS

This paper was written, reviewed and approved by all four authors.

## ETHICS STATEMENT

The ethics statement is not applicable for this manuscript.

### PEER REVIEW

The peer review history for this article is available at https://publons.com/publon/10.1111/nan.12757.

## Supporting information


**Table S1.** Summary of differentially methylated genes that have been identified in two or more studies, both within an NMD or between different NMDs, in case–control comparisons.

## Data Availability

Data sharing is not applicable to this article as no data sets were generated or analysed during the current study.
